# Unravelling Sleep‐Pain Interactions: Methodological Advantages and Inflammation

**DOI:** 10.1002/ejp.70438

**Published:** 2026-10-23

**Authors:** Stefan Lautenbacher

**Affiliations:** ^1^ Department of Medical Psychology and Sociology University of Augsburg Augsburg Germany

This journal recently published a paper by the research group of Monika Haak entitled ‘Effects of low‐dose acetylsalicylic acid on spontaneous pain, pain sensitivity, and central pain modulation in response to a sleep restriction challenge in healthy individuals’ (Nett et al. 2026). The study builds on the well‐established bidirectional relationship between sleep disturbances and pain, in which each can exacerbate the other. The authors hypothesized that inflammatory processes may be key mediators of this relationship. In a randomized, double‐blind crossover study, they investigated whether pre‐emptive administration of low‐dose acetylsalicylic acid (ASA; aspirin) could mitigate the pain‐enhancing effects of sleep restriction in healthy adults. Sleep restriction involved five consecutive nights with only 4 h of sleep opportunity. Although 14 days of ASA administration did not affect baseline pain ratings, pain sensitivity, or pain modulation, it attenuated the increase in widespread and muscle pain associated with sleep restriction and reduced the impairment of endogenous pain inhibition compared with placebo. These findings suggest that inflammatory pathways may contribute to the effects of insufficient sleep on pain processing.

The merit of this study rests on two major achievements: (1) the methodological excellence of experimentally simulating everyday sleep disturbances through repeated sleep restriction and (2) the opportunity to not only investigate the relationship between disturbed sleep and pain but also to experimentally modify their pathological linkage, thereby gaining insights into the underlying mechanisms.

Natural variations in sleep among healthy individuals are not always sufficient to demonstrate reliable changes in the pain system. More pronounced effects can be observed when sleep is experimentally restricted or fragmented (Lautenbacher 2018). The research group of Monika Haak has developed excellent experimental designs and protocols with high ecological validity that produce sufficiently robust and reproducible effects on pain processing. The partial sleep restriction paradigm used in the present study was designed to reflect common patterns of insufficient sleep during the working week, followed by recovery sleep on weekends, as frequently observed in modern societies. Developing such ecologically valid paradigms is essential to model the transition from a at first relatively balanced pain system to one increasingly affected by initially subclinical and potentially later clinically relevant sleep disturbances (Simpson et al. 2018).

The mechanisms underlying the relationship between sleep disturbances and pain remain largely unknown. One reason for that is the considerable time, effort, and resources required to design and conduct studies capable of identifying specific mechanisms. The present study illustrates these challenges, requiring substantial commitment from participants and experimental staff to collect the necessary data. The associated costs and logistical demands mean that only specialized research centres can conduct such investigations. The research group of Monika Haak deserves recognition for establishing the methodological expertise and infrastructure necessary to undertake this demanding work.

Against the background of these enormous methodological achievements, the choice to investigate the effects of the long‐established drug ASA at a dose below its conventional analgesic range might initially seem surprising. However, this proved to be a thoughtful decision. The observed attenuation of sleep restriction‐induced pain suggests that inflammatory pathways may contribute to the sleep–pain relationship. Although such pathways have long been suspected, the present findings provide firm experimental support for their involvement. Nevertheless, the precise inflammatory mechanisms remain to be established.

The findings also open up promising avenues for further research. Given the widespread use of low‐dose ASA in certain age groups and clinical populations, epidemiological studies could investigate whether the association between sleep disturbances and pain differs between ASA users and non‐users while carefully accounting for potential confounding factors. Similarly, clinical studies in patients with insomnia could examine the temporal relationship between sleep disturbances and clinical pain, exploring whether early targeting inflammatory pathways modifies this interaction.

In conclusion, the study by Nett et al. demonstrates how experimentally controlled and ecologically relevant sleep restriction paradigms can be combined with pharmacological interventions to investigate the mechanisms linking disturbed sleep and pain. It represents an important step towards clarifying the role of inflammation in this relationship and provides a valuable foundation for future research into the specific pathways through which insufficient sleep affects pain processing.

## Linked Articles

This article is linked to Nett et al. papers. To view this article, visit https://doi.org/10.1002/ejp.70392.
